# Wengen, the Sole Tumour Necrosis Factor Receptor in *Drosophila*, Collaborates with Moesin to Control Photoreceptor Axon Targeting during Development

**DOI:** 10.1371/journal.pone.0060091

**Published:** 2013-03-27

**Authors:** Wenjing Ruan, Nicolas Unsain, Julie Desbarats, Edward A. Fon, Philip A. Barker

**Affiliations:** Centre for Neuronal Survival, Montreal Neurological Institute, McGill University, Montreal, Quebec, Canada; University of Massachusetts Medical School, United States of America

## Abstract

Photoreceptor neurons (R cells) in the *Drosophila* eye define a map of visual space by connecting to targets in distinct layers of the optic lobe, with R1-6 cells connecting to the lamina (the first optic ganglion) and R7 and R8 cells connecting to the medulla (the second optic ganglion). Here, we show that Wengen (Wgn) directly binds Moesin (Moe) through a cytosolic membrane proximal domain and this interaction is important for mediating two distinct aspects of axonal targeting. First, we show that loss of *wgn* or *moe* function disrupts cell autonomous R8 axon targeting. Second, we report that *wgn* or *moe* mutants show defects in R2–R5 targeting that result from disruption of non-cell autonomous effects, which are secondary to the cell autonomous R8 phenotype. Thus, these studies reveal that the Wgn-Moe signaling cascade plays a key role in photoreceptor target field innervations through cell autonomous and non-cell autonomous mechanisms.

## Introduction

Determining the molecular basis of neuronal targeting and identifying the mechanisms that lead to the establishment of synaptic circuits is a critical issue in neurobiology. Neurons develop and extend processes in a stepwise and stereotypical fashion and it is certain that short- and long-range guidance cues can attract or repel growth cones, and facilitate or inhibit synapse formation [1], [2]. However, our knowledge of the specific mechanisms that allow developing neurons to seek out appropriate target zones and form synapses remains incomplete.

The compound eye of *Drosophila* contains ∼ 800 ommatidia, each of which has 3 types of R cells (R1-6, R7 and R8) [3], [4]. During larval development, the R8 photoreceptor differentiates earliest and is the first to extend its axon into the optic lobe, followed by R1-6 and R7. The R1-6 and R7 axons fasciculate with the R8 axons and grow along R8 to reach their target zones. The R1-6 growth cones follow R8 axons only to the lamina where they terminate. R7 growth cones follow R8 axons through the lamina and into the medulla and terminate into a deeper layer (M6).

In mammals, tumor necrosis factor receptor (TNFR) superfamily members mediate a wide spectrum of physiological and pathological events. Interestingly, recent studies have indicated that TNFR superfamily members regulate morphogenetic activity [5], with FAS, DR6, and p75^NTR^ playing important roles in neuronal process outgrowth and integrity [6]–[8]. We have characterized some of the intracellular pathways that regulate FAS-mediated process outgrowth in primary mammalian cortical neurons and shown that a direct interaction between FAS and Ezrin, an ERM (Ezrin, Radixin, Moesin) family member, is required for this function [9]. However, characterizing the fundamental physiological relevance of this pathway in mammals *in vivo* is complicated by the existence of numerous compensatory pathways. *Drosophila* has a single TNFR-like receptor, termed Wgn [10], [11] and one TNF-like ligand, termed Eiger (Egr) [11]–[13]. In fly, the only one ERM, Moe, is present [14], [15]. The expression of single TNFR and ERM makes *Drosophila* a tractable system for examining their functions and signaling mechanisms *in vivo*.

Here, we report that Wgn is expressed in R cells and is required for appropriate targeting of both R8 and R2–R5 axons. Interestingly, Wgn and Moe mediate their effects on R8 and R2–R5 targeting independent of Egr, the TNF-like ligand. Wgn binds directly to Moe and the Wgn-Moe cassette functions in a cell autonomous manner to mediate effects on R8 path finding. However, the effect of Wgn and Moe on R2–R5 axons depends entirely upon non-cell autonomous actions derived from R8. Thus, Wgn and Moe function within R8 cells to control axon guidance and targeting during development.

## Materials and Methods

### Drosophila Stocks and Genetic Crosses

**Figure 1:**
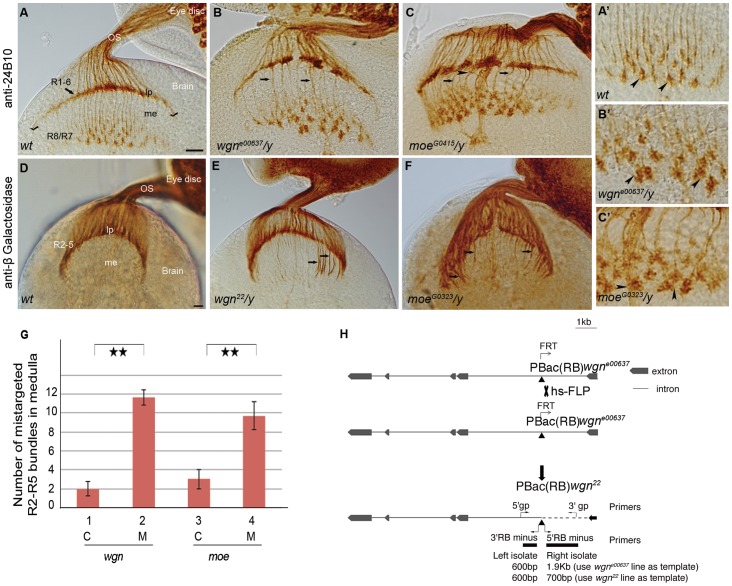
*wgn* or *moe* mutation results in R cell axonal targeting defects. (A–C) Eye-brain complexes of third-instar larvae were stained with 24B10 to visualize all R cell axons. (D–F) Samples were labeled with *Ro-tau-LacZ* marker and stained by anti-beta galactosidase to visualize R2–R5 axons. (A′–C′) Magnified view of medullar areas in A–C. (A) In wild-type flies, R1-6 growth cones terminated in the lamina to form the lamina plexus (seen as a smooth line, stretching between two check marks in A), while R7 and R8 axons projected into the medulla. R7 and R8 growth cones expanded to form a regular array of non-overlapping growth cones (also see magnified view in A′). In *wgn^e00637^/y* (B) and *moe^G0415^/y* (C) mutants, the lamina plexus was disrupted and the termination fields in the medulla were disorganized and contained many thick wandering bundles (arrows). The growth cones within the medulla were enlarged and overlapped (arrow heads in B′ and C′). (G) Quantifications of mistargeted R2–R5 axon bundles projected into medulla in control, *wgn* and *mo*e mutant animals. Double stars: P < 0.001; Student T tests, two tails. Error bars denote s.e.m. Genotypes in (G)*:* #1(*wgn^22^/+*; *rtl*/+); #2 (*wgn^22^*/*y*; *rtl*/+); #3 (*moe^G0323^*/+; *rtl*/+); #4 (*moe^G0323^*/*y*; *rtl*/+). (H) A diagram illustrating the generating of *wgn^22^* mutant. The P element insertion site in the *wgn^e00637^* strain is located in the first intron of *wgn* at X:18526488 in the minus orientation (indicated by the point upward triangle). FRT-mediated recombination of the *wgn^e00637^* strain resulted in production of the *wgn^22^* recombinant line in which the P element remained but deletion of approximately 1200 base pairs was introduced. Abbreviations: lp, lamina plexus; me, medulla; OS, optic stalk; *rtl*, *Rough-tau-LacZ*; C, control; M, mutant; gp, genomic primer; hs-FLP, heat shock flippase. Scale bars: A–C, 10 µm; D–F, 10 µm.

w^1118^, wgn^e00637^, Df(1)E128/FM7c, moe^G0323^/FM7c, moe^G0415^/FM7c, moe^EP1652^/FM7c, UAS-moe-Myc, UAS-moeT559D-Myc, ey-FLP,Sb/TM6B,Tb, and FRT 19A, Tub-Gal80, hsFLP; mCD8-GFP lines were provided by Bloomington Drosophila Stock Center. UAS-wgn-RNAi (GD3427^V9152^), UAS-moe-RNAi (GD5211^V37917^), UAS-pink1-RNAi (GD11336^V21860^) and UAS-grip-RNAi (GD14152^V29073^) lines were provided by Vienna Drosophila RNAi Center. egr^e02904^ was provided by Exelixis collection at Harvard University. The insc^22^, egr^66^ was provided by X. Yang [16] and the Gal4^109–68^ line was provided by Y. Rao ((McGill University) (original from [17], [18]). The wgn^22^ allele was generated by FRT-mediated homologous recombination that involved crossing two sister chromosomes containing piggyBac elements inserted into first intron of wgn with heat shock flippase (hs-FLP) as source of flippase [19]. From over 100 recombinant lines, one line had a different 5′ region from the original P insertion site and this was named wgn^22^ (See detail in Figure 1J). For MARCM analysis, we used hs-FLP together with GMR-Gal4 driver, Ro-tau-LacZ and UAS-mCD8-GFP markers to generate mutant or wt cells which were labelled with GFP and with Ro-tau-lacZ [20]. In order to identify R8, we used anti-Senseless to stain R8 cells. We traced the trajectories of the R8 cells that were co-stained with anti-Senseless in R8 nuclei and anti-GFP in cell bodies and axons from eye disc to optic lobe. We noticed that GFP proteins were expressed in Bolwig’s nerve in all the samples examined regardless of the presence of the transgene Tub-Gal80. To generate UAS-wgn-Flag and UAS-wgn-ΔMPD-Flag transgenic flies, wgn cDNA was obtained from the full length EST clone RE29502 (Berkeley Drosophila Genome Project). Full length and deleted MPD domain of Wgn were generated by PCR-based procedures and subcloned into pUAST vector. The transgenic flies were generated by Best Gene Inc.

### Plasmid Constructs, Protein Purification and Antibody Production

pMal-C2X (New England Biolab) and pGEX-4T-1 (GE) was used to express MBP-ΔECD, GST-ΔECD and GST-ΔECD-ΔMPD in E. coli strain BL21. MBP or GST fusion proteins were purified from bacterial lysates using appropriate affinity column. The GST-ΔECD protein was purified and used to produce anti-Wgn sera in rabbits. The antibody was affinity purified by MBP-ΔECD protein that was affixed to PDVF membrane and eluted using 50 mM glycine pH 2.5. After adjusting to pH 7.0, the buffer was exchanged into phosphate-buffered saline using Amicon Ultracentrifugal Filter Unit with a 10 KDa molecular weight cut-off (Millipore).

### In vitro Binding Assay

In vitro binding assay was performed as described [21]. The anti-Myc monoclonal antibody 9E10 (Santa Cruz) was used to detect Myc tagged Moe.

### Immunohistochemistry

Whole mount eye-brain complexes of third-instar larva were prepared as described [22]. R1-8 cell axons were labelled with the 24B10 monoclonal antibody [23]. The R2–R5 axons were labelled with *Ro-tau-LacZ,* as described [24] whereas R8 axons were labeled with *Ato-tau-Myc* [25], [26]. The monoclonal antibodies 24B10 (1∶200), mouse anti-beta galactosidase (40-1a) (1∶1000), mouse anti-Prospero (1∶200), mouse anti-Elav (1∶200), mouse anti-Dachshund (1∶200) and mouse-anti-Repo (1∶200) were from the Developmental Studies Hybridoma Bank. The guinea pig anti-Senseless [27] (1∶2000) was provided by HJ Bellen. Rabbit anti-GFP (1∶2000) was from Invitrogen. The secondary antibodies were goat anti-mouse-HRP (1∶200), goat anti-rabbit-HRP (1∶200) (Bio-Rad), Alexa 488-conjugated goat anti-rabbit (1∶2000), Alexa 594-conjugated goat anti-mouse (1∶2000), Alexa 633-conjugated goat anti-guinea pig (1∶2000) (Molecular Probes), and Cy3-conjugated goat anti-mouse (1∶1000) (Jackson ImmunoResearch). The bright field images were taken on a Nikon Eclipse E800 microscope. The fluorescent images were captured on a Zeiss LSM-510 or 710 confocal microscopies.

## Results

### Wgn and Moe are Required for R Cell Axon Targeting

We initiated our examination of the function of Wgn in R cell axon guidance by staining third-instar larval eye-brain complexes using 24B10 [23], an R cell-specific monoclonal antibody. R1-6 axons normally project towards the posterior aspect of the eye imaginal disc, pass through the optic stalk, then fan out and terminate in the first optic ganglion, where they form the lamina plexus [3], [4]. R7 and R8 axons take the same route but project beyond the lamina plexus to terminate in the medulla. In this medullar region, termed the second optic ganglia, the growth cones of R7 and R8 normally form evenly spaced arrays of “inverted-Y shaped” structures (Figure 1A). In flies lacking functional Wgn, innervation of the lamina and medulla was clearly disrupted. Figure 1B shows that in *wgn^e00637^* mutants (which have a piggyBac element inserted in the first intron of the *wgn* gene), the normal smooth structure of the laminar plexus is replaced by large aggregates separated by gaps (compare Figures 1A and 1B). In the medulla, *wgn^e00637^* mutants display abnormally thick axon bundles and medullar axons often strayed into neighbouring regions (arrows in Figure 1B). Furthermore, growth cones in the medulla were enlarged and often overlapped with each other (compare arrows in Figure 1A′ and 1B′).

The *wgn^e00637^* phenotype was partially penetrant (70%; n = 27), perhaps because this strain is a *wgn* hypomorph [19]. To produce a more complete loss-of-function allele, we disrupted the 5′ region adjacent to the original P-element insertion site in *wgn^e00637^* using FRT-mediated homologous recombination (Figure 1). The phenotype in the resulting *wgn^22^* strain was fully penetrant (100%; n = 14) and more severe than in *wgn^e00637^*, with many large gaps in the lamina and with numerous abnormal thick bundles projecting into the medulla (Data not shown and Figure 1).

We speculated that the thick axon bundles entering the medulla in *wgn* mutants were abnormal projections of R1-6, which normally terminate in the lamina. To address this, we crossed *wgn^22^* with the *Ro-tau-lacZ* marker line, which identifies R2–R5 axons (Table 1) [24]. In wild-type flies, R2–R5 axons usually terminate in the lamina but in *wgn^22^* flies, many R2–R5 projections fail to terminate in the lamina but instead, grow further to enter the medulla (penetrance 100%, n>10). Quantification of the mis-entry of R2–R5 axons into the medulla in *wgn^22^* flies revealed a highly significant increase in abnormal R2–R5 projections compared to wild-type (Figure 1). The *wgn^e00637^* strain, the *Df(1)E128/y* strain (in which several genes including *wgn* were deleted) and the *wgn^22^*/*Df(1)E128* strain showed very similar phenotypes (Data not shown). We conclude that the TNFR superfamily member Wgn is required for normal termination of R2–R5 axons in the lamina.

**Table 1:** Gal4 Drivers and Markers Used in This Study.

Name of fly lines	Expression patterns	References
*Ro-tau-LacZ (rtl)*	β-Galactosidase expression in R2–R5 axons	24
*Ato-tau-Myc(atm)*	Myc protein expression in R8 axon and growth cones	25,26
*109.68-Gal4*	Gal4 protein expression in R8 cells	17,18
*puc-LacZ*	β-Galactosidase expression driven by *puckered* promoter	32
*GMR-Gal4*	Gal4 expression in the eye	30
*Elav-Gal4*	Gal4 expression in neurons	31

In mammalian neurons, the adaptor protein Ezrin, a member of the ERM family, links the TNFR family member FAS receptor to the cytoskeleton to propel neurite outgrowth *in vitro* [9]. Our observation of the R cell axon targeting function of Wgn prompted us to test whether the single ERM family protein in fly, Moe, plays a role in Wgn-dependent R cell axon targeting. To address this, we first examined R cell projection patterns in third-instar larvae homozygous for *moe^G0415^* and *moe^G0323^,* well characterized *moe* loss-of-function alleles [28]. Figure 1C shows that *moe^G0415^* flies had R cell projections defects that were qualitatively identical to those observed in *wgn* mutants. Specifically, the *moe^G0415^* flies had gaps in the laminar plexus, displayed thick axon bundles that projected into medulla (arrows in Figure 1C), had abnormal wandering projections (arrow head in Figure 1C), and had medullar growth cones that were enlarged and overlapping (compare arrow heads in Figure 1A′ and 1C′). Using the *Ro*-*tau-lacZ* marker, we found that *moe^G0323^* R2–R5 axons did not terminate in the lamina but instead projected into the medulla (Figure 1F and Quantified in 1G). The *moe^G0323^* phenotypes were 100% penetrant (n = 30) and these phenotypes were observed in flies with other *moe* alleles, including *moe^G0415^, moe^EP1652^* and *moe^G0415^/moe^EP1652^* trans-heterozygotes (Data not shown).

**Figure 2:**
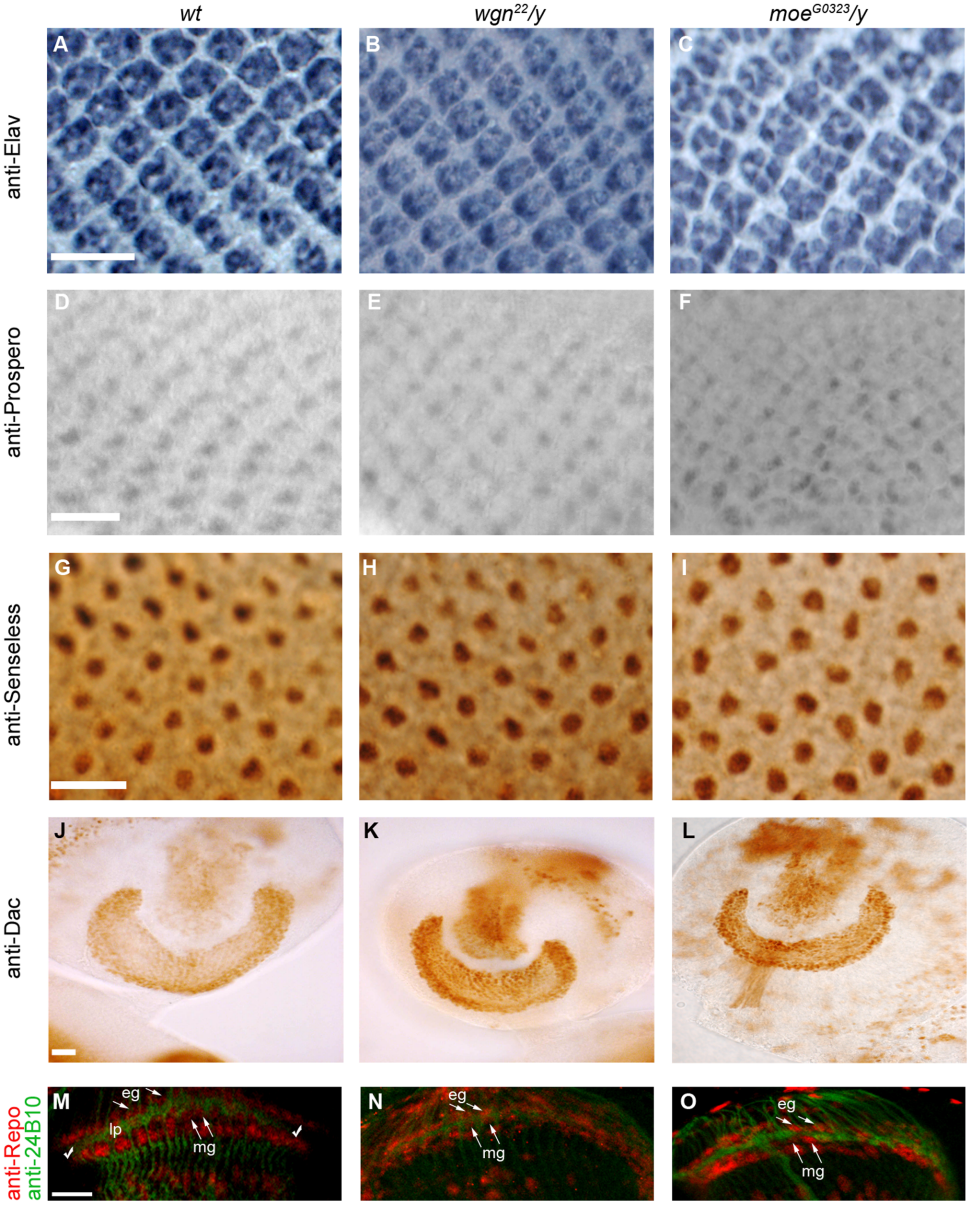
R cell fate, lamina neuron development, and glia cell development are normal. Eye discs stained with anti-Elav (A–C), anti-Prospero (D–F), and anti-Senseless (G–I) showed normal staining pattern for all R cells, R7 and R8, respectively. Brains stained with anti-Dachshund (J–L) showed normal differentiation of lamina neurons. (M–O) Eye-brain complexes double stained with 24B10 (green) for R cell axons and anti-Repo (red) for glial cells. Lamina plexus (green, between two check markers) was present between two layers of glial cells (red), epithelial glia and marginal glia. The number of glial cells in R1-6 target region in *wgn* and *moe* mutants was similar to wild-type. This indicated that the differentiation and migration of glial cells were normal in *wgn* and *moe* mutants. The array of glia around lamina plexus was mildly disorganized in both mutants. This was likely caused by abnormal projection of R cell axons. Arrows indicate glial cells. Abbreviations: lp, lamina plexus; eg, epithelial glia; mg, marginal glia. Scale bars: A–C, D–F, and G–I, 10 µm; J–L and M–O, 20 µm.

To rule out the possibility of the observed R cell axon targeting defects are secondary to abnormal R cell, lamina neuron or glial cell differentiations, we examined their differentiation with several cell markers (anti-Elav for all R cells, anti-Prospero for R7, anti-Senseless for R8, anti-Dachshund for lamina neurons, and anti-Repo for glial cells). In each case, the staining patterns for *wgn* and *moe* mutants were normal, like the wild-type (Figure 2). We conclude that *wgn* or *moe* phenotypes reflect primary defects of axon guidance and targeting rather than secondary defects resulting from defects in cell differentiation or fate.

### Wgn is Present in R Cells

**Figure 3:**
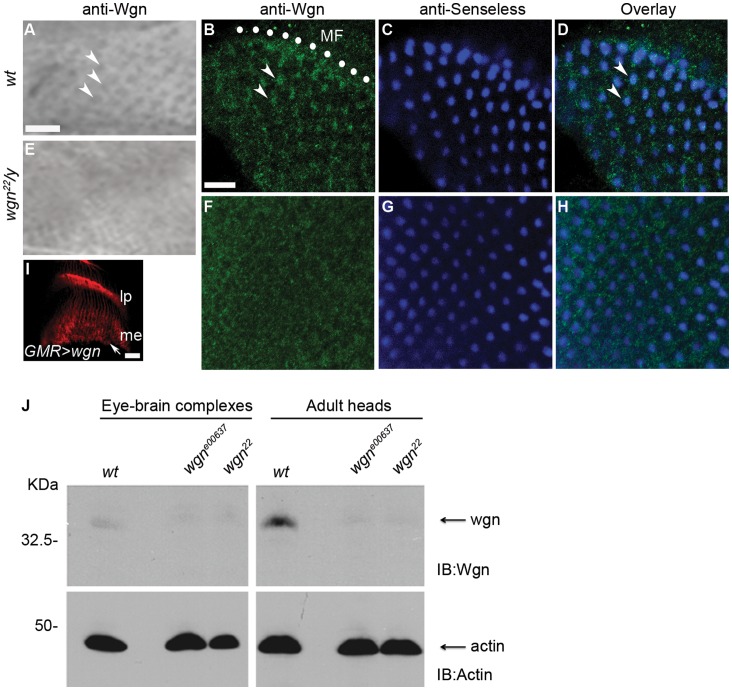
Wgn proteins are expressed in R cells, especially in R8. (A–D) Wild-type. (E–H) *wgn* mutant. (A and E) Third-instar eye-brain complexes were stained with anti-Wgn. Wgn proteins were expressed in R cells (viewed as regular array of dots indicated by arrows) in wild-type animal (A) but not in *wgn* ^22^ */y* mutant (E). (B–D and F–H) Third-instar eye-brain complexes were double stained with anti-Wgn (green in B and F) and anti-Senseless (blue in C and G). (B–D) In wild-type, Wgn was present in the Senseless-expressing R8 cells (indicated by arrow heads in B and D) in the region posterior to the morphogenetic furrow (MF, denoted by white dots in B). (F–H) Wgn protein in R8 cells was absent in *wgn* mutant (*wgn^22^/y*). (I) When overexpressed in the eye, Wgn protein was transported into axons and growth cones (arrow) in the brain. Genotype: *w^1118^*; *GMR-Gal4*/+; *UAS-wgn-Flag*/+. (J) Immunoblot of lysates of eye-brain complexes or adult heads from indicated strains showed levels of Wgn. Wgn proteins were detected in wild-type but not in *wgn* mutant animals.

Previous studies have shown that Moe protein is present in the cell bodies of all R cells [29] but the endogenous expression pattern of Wgn has not been determined. To address this, we generated an anti-Wgn antibody and used it to stain third-instar larval eye-brain complexes. In the wild-type eye disc, anti-Wgn antibody stained R cell bodies as a regular array of dots (indicated by arrow heads in Figure 3A) whereas it did not stain *wgn^22^* mutant eye discs. Furthermore, Wgn protein was present in cells that were co-stained with Senseless, most prominently within newly forming ommatidia near the morphogenetic furrow (arrow heads in 3B), indicating that Wgn is expressed in R8 cells (Figure 3B–3D). This R8 staining is absent in *wgn^22^* mutant animals (Figure 3F–3H). Within wild-type eye-brain complexes, axonal Wgn expression was below our antibody detection limit but the protein was readily detected in axons and growth cones when *GMR-Gal4* [30] was used to drive overexpression of Wgn proteins in the eye discs (arrow in Figure 3I), indicating that Wgn can be transported to these cellular domains. By immunoblotting, we found that endogenous Wgn expression was present at low but detectable levels in wild-type third-instar eye-brain complexes and at higher levels in adult heads; Wgn protein was not detectable in *wgn^e00637^* or *wgn^22^* flies (Figure 3J).

### Mutations in wgn and moe cause R8 Growth Cone Morphology Defects

**Figure 4:**
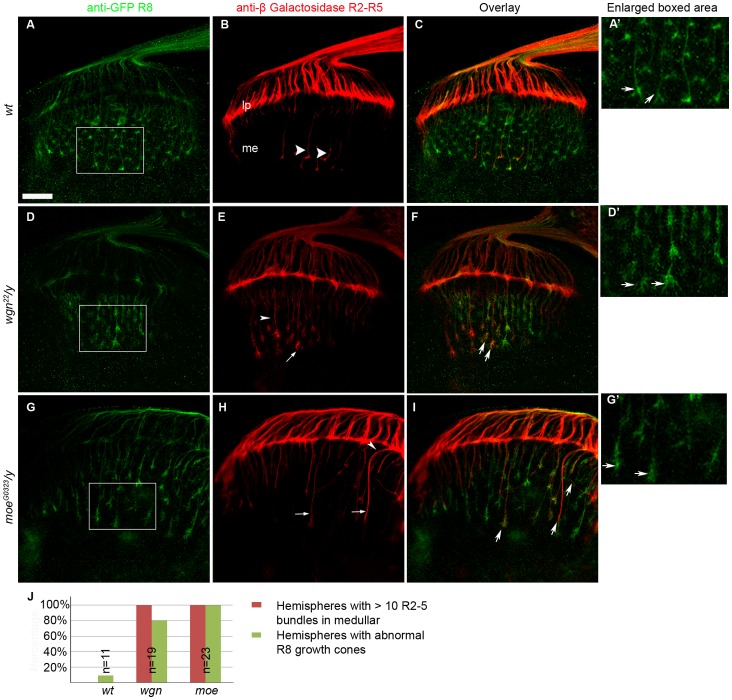
Mutations in *wgn* and *moe* result in defects in R8 growth cone morphology. (A–C) Wild-type. (D–F) *wgn* mutant. (G–I) *moe* mutant. In panels A, D, and G, anti-Myc stained all R8s. The *wt* R8 growth cones formed a regular array of “inverted-Y shaped” structures (A and arrows in A′). This normal R8 morphology was disrupted in *wgn* (D and arrows in D′) and *moe* (G and arrows in G′) mutant animals. In panels B, E, and H, R2–R5 axons were visualized by anti-beta galactosidase staining. Few *wt* R2–R5 axons around the middle region of lamina plexus projected through and terminated in the medulla (arrow heads in B) whereas entry of R2–R5 axons into the medulla was prevalent in *wgn* (E) and *moe* (H) mutants (indicated by arrows and arrow heads). Note that mistargeted R2–R5 axons were often followed R8 paths (indicated by arrows in F and I). Quantifications of the R8 growth cone phenotypes and R2–R5 axon mistargeting were shown in Panel J. Hemispheres with > 10 R2–R5 axons and/or with thicker bundles in the medullar region were counted as hemispheres with R2–R5 mistargeting defects. Hemispheres that have irregular array of R8 growths cones in the medullar region with blunt or enlarged ends were counted as hemispheres with abnormal R8 growth cones. Genotypes: in A–C and A′, *w^1118^*; *rtl*, *atm/+*; in D–F and D′, *wgn^22^*/*y*; *rtl*, *atm*/+; in G–I and G′, *moe^G0323^*/*y*; *rtl*, *atm*/+; in J: *wt* (*w^1118^*; *rtl*, *atm/+*); *wgn* (*wgn^22^*/*y*; *rtl*, *atm*/+); *moe* (*moe^G0323^*/*y*; *rtl*, *atm*/+). Abbreviations: lp, lamina plexus; me, medulla; *rtl*, *Rough-tau-LacZ; atm*, *Atonal-tau-Myc*. Scale bar: A–I, 10 µm.

To ask whether *wgn* mutants had a specific R8 phenotype, R8 cells that were marked by *Ato-tau-Myc* [25], [26] were crossed into the *wgn^22^* background. In wild-type animals, R8 growth cones form a regular array of “inverted-Y shaped” structures (Figure 4A and 4A′) but these were disrupted in *wgn^22^/y* mutants. Many of the R8 growth cones in the *wgn^22^/y* mutants were enlarged and had numerous fine extensions but others were constricted and had blunt ends (Figure 4D and 4D′). We also crossed *wgn^22^/y* to *Ro-tau-LacZ* to specifically mark R2–R5 cells. As expected, R2–R5 axons in the *wgn^22^/y* strain showed extensive mistargeting into the medullar region (arrows and arrow head in Figure 4E and 4F). Interestingly, a similar array of R8 growth cone phenotypes was also observed in *moe^G0323^/y* mutants (Figure 4G and 4G′). *moe^G0323^/y* mutants that had been crossed to *Ro-tau-LacZ* revealed qualitatively similar R2–R5 mistargeting phenotypes (arrows and arrow head in Figure 4H and 4I). Notably, in both *wgn* and *moe* mutant animals, mistargeted R2–R5 axons often followed the paths of R8 axons into the medullar region (arrows in Figure 4F and 4I). Quantification and statistical analyses of these defects are provided in Figure 4J.

### Wgn and Moe Interact Physically and Genetically

**Figure 5:**
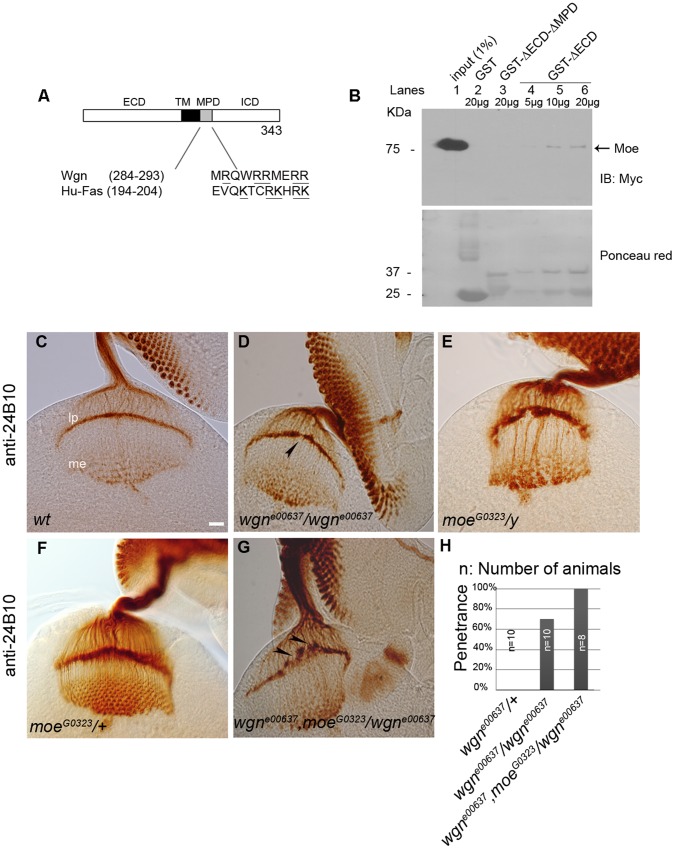
Wengen and Moesin interact physically and genetically. (A) Alignment of MPD domain in Wgn and human Fas, with clusters of basic amino acids underlined. (B) Lysates prepared from fly heads (*w^1118^*; *GMR-Gal4*/*UAS-moe-Myc*) were used in the pull down assays with immobilized GST, GST-ΔECD-ΔMPD, or GST-ΔECD. Pull downs were analyzed by anti-Myc immunoblot (upper panel). The amount of GST, GST-ΔECD-ΔMPD and GST-ΔECD proteins used for pullouts were shown by Ponceau red staining (lower panel). (C–G) Eye-brain complexes were stained by 24B10. R cell axon projections were normal in wild-type (C) and *moe^G0323^*/+ (F) heterozygous animals. (D) *wgn^e00637^* mutant. Lamina plexus was disrupted. It appears uneven and has a gap indicated by arrow head. (G) Removing one copy of *moe^G0323^* enhanced the *wgn^e00637^* phenotype with large gaps apparent in the lamina plexus (arrow heads). (H) Quantifications of genetic interactions. By removing one copy of *moe* in *wgn^e00637^* mutant background, the penetrance of *wgn^e00637^* increased from 70% to 100%. Scale bar, 10 µm.

We previously showed that in mammalian cells, the ERM family member Ezrin binds to a juxtamembrane domain in FAS termed the MPD [9]. The phenocopy of *wgn* and *moe* suggests that these two molecules may function in the same pathway to control R cell axon targeting. Comparison of FAS and Wgn amino acid sequences revealed that an MPD-like sequence is present in the juxtamembrane region of Wgn (Figure 5A) and we therefore tested whether Wgn and Moe can directly interact. Wgn-GST fusion proteins that contained (GST-ΔECD) or lacked (GST-ΔECD-ΔMPD) the putative MPD were examined for their ability to bind Moe present in fly lysates derived from adult heads that expressed *UAS-moe-Myc* transgene by using *GMR-Gal4* driver. Figure 5B shows that the GST-ΔECD bound Moe in a dose-dependent manner whereas GST-ΔECD-ΔMPD did not. We conclude that Wgn binds to Moe and that the MPD domain within Wgn is required for this association.

To determine if the interaction of Wgn with Moe is relevant *in vivo*, we asked whether the incidence of R cell axon mistargeting defects is changed when the *moe* gene dosage is reduced in a *wgn* hypomorphic (*wgn^e00637^*) background. Reducing the *moe* gene dosage by half had no consequence in otherwise wild-type flies but enhanced axonal mistargeting defects in *wgn^e00637^* mutant background. In these, the lamina plexus was severely disrupted and frequent gaps and thick bundles projected into the medullary region (compare Figure 5D and Figure 5G). The penetrance of the *wgn^e00637^* mutant phenotype increased from 70% to 100% when the *moe* gene dosage was halved (Figure 5H). Taken together with the physical association of Wgn and Moe, these data support the notion that Wgn and Moe function together in a signaling pathway that is crucial for normal R cell axon guidance and targeting.

### R Cell Axonal Targeting Relies on Specific Levels of Wgn and Moe Expression

**Figure 6:**
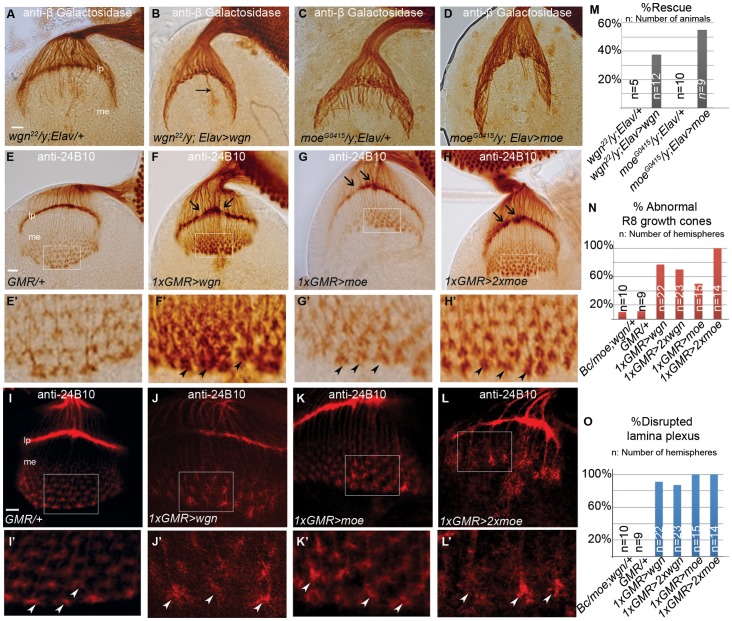
Neuronal expression of Wgn or Moe partially rescues mutant phenotypes and overexpression of Wgn or Moe in R cells disrupts R cell axonal targeting. (A–D) Eye-brain complexes of third-instar larvae were labeled with a *Ro-tau-LacZ* marker and stained with the anti-beta galactosidase antibody. (E–L) R cell axons of third-instar larvae were stained with 24B10. (E′–L′) Enlarged boxed areas in E–L. (A) Many R2–R5 axons and axonal bundles were present in the medulla of *wgn* mutants (*wgn^22^*/*y; rtl/+*). (B) Expression of a *UAS-wgn-Flag* transgene from an *Elav-Gal4* driver rescues R2–R5 axon mistargeting in 37.5% individuals (*wgn^22^*/*y; Elav-Gal4/+; rtl/UAS-wgn-Flag*). The arrow in (B) indicates the Bolwig’s nerve, which is a larval photosensitive structure that is also marked by *Ro-tau-LacZ.* (C) Numerous R2–R5 axons and bundles projected into the medulla in *moe* mutants (*moe^G0415^/y*; *rtl*/+) and (D) expression of a *UAS-moe-Myc* transgene from an *Elav-Gal4* driver in *moe* mutants rescued these mistargeting events in 55% of individuals (*moe^G0415^/y*; *Elav-Gal4, UAS-moe-Myc/+; rtl*+). (E and I) Wild-type larvae (*GMR-Gal4/+*). (F and J) Larvae carrying one copy of *GMR-Gal4* and *UAS-wgn-Flag* transgene (*w^1118^*; *GMR-Gal4/+; UAS-wgn-Flag*/+). (G and K) Larvae carrying one copy of *GMR-Gal4* and *UAS-moe-Myc* transgene (*w^1118^*; *GMR-Gal4/UAS-moe-Myc*). (H and L) Larvae carrying one copy of *GMR-Gal4* and two copies of *UAS-moe-Myc* (*w^1118^*; *GMR-Gal4, UAS-moe-Myc/UAS-moe-Myc*). (E′–L′) Magnified view of boxed areas in E–L showed that growth cones in medullar region were expanded and overlapped when Wgn (F′ and J′) or Moe (G′–H′ and K′–L′) proteins were overexpressed. (M–O) Quantifications of rescue (shown in A–D) and overexpression (shown in E–L) experiments. Abbreviations: lp, lamina plexus; me, medulla; *rtl*, *Rough-tau-LacZ*. Scale bars: A–D, 10 µm; E–H, 10 µm; I–L, 10 µm.

To determine if the R cell axon targeting defects could be rescued, we used the neuronal specific driver *Elav-Gal4* [31] to drive expression of a *UAS-wgn* transgene in a *wgn^22^* mutant. This resulted in rescue of R cell axon mistargeting defects in 37.5% of individuals (n = 12; Figure 6A and 6B), a relatively modest effect. Partial rescues were also obtained when *Elav-Gal4* was used to drive expression of a *UAS-moe* transgene in the *moe^G0415^* strain, with 55% of individuals displaying rescue of the R cell axon mistargeting defects (n = 9; Figure 6C, 6D). One explanation for these partial rescues is that R cell axon projection patterns rely on a narrow window or specificity of Wgn and Moe expression. Consistent with this, we found that in otherwise wild-type flies, expression of Wgn or Moe by *GMR-Gal4* resulted in R cell axon projection defects identical to those observed in the loss-of-function mutant animals (Figure 6E–L, E′–L′ and 6N–6O). Therefore, it seems that transgenic expression of Wgn or Moe can cause a rescue in some individuals but in others, exceeds an expression threshold that disrupts R cell axon targeting. If this is the case, dampening transgene expression should increase the proportion of rescued individuals. Consistent with this, when flies were reared at a lower temperature (18 °C) to reduce *UAS-wgn* transgene expression, the proportion of rescued animals increased from 37.5% to 50% (n = 14). These data suggest that normal R cell axon targeting requires a narrow range of Wgn and Moe expression. Since previous studies have shown that overexpression of Egr using the *GMR-Gal4* driver induces JNK activation and cell death [12], [13], we also asked whether Wgn overexpression activates JNK in R cells. Interestingly, we found that Egr overexpression mediated by *GMR-Gal4* readily activated *puc-LacZ* (a transgene that indicates JNK activation [32]) and caused massive R cell death. In contrast, *GMR-Gal4* driven Wgn expression did not induce *puc-LacZ* expression, R cell loss or alter eye morphology (data not shown).

### Cell Autonomous Axonal Targeting Defects Occur only in R8 Cells

**Figure 7:**
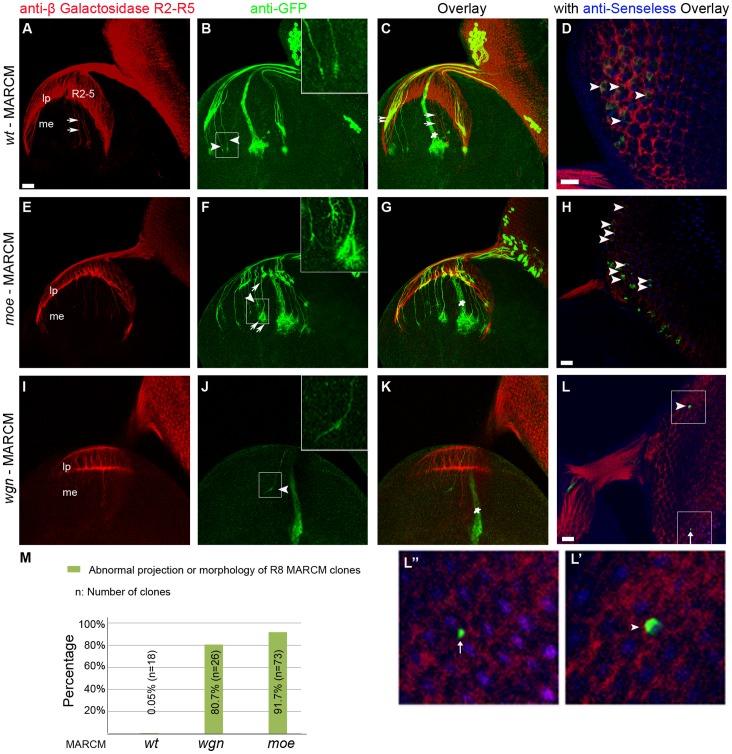
Wgn and Moe function cell autonomously to mediate R8 axon targeting. MARCM analysis of *wt*, *wgn* and *moe* clones. MARCM clones were induced by heat shock flippase (hs-FLP) and labeled by GFP (Green). R2–R5 cells were marked by *Rough-tao-LacZ* and stained by anti-beta galactosidase (red). R8 cells were stained by anti-Senseless (blue). (A–D) With wild-type, R2-5 growth cones (red) terminated at the lamina plexus (A) but a few R2-5 axons surrounding the Bolwig’s nerve (indicated by white star in C) passed through the lamina and terminated into medulla (white arrows in A and C). Note that the GFP proteins were expressed in Bolwig’s nerve in all the samples examined regardless of the presence of the transgene Tub-Gal80. The axons of wild-type R2–R5 clones terminated into lamina plexus (double arrow heads in C) whereas axons of wild-type R8 MARCM clones projected into the medulla without turning into adjacent neighboring regions (arrow heads in B) in which they displayed condensed growth cones (enlarged insert in B). (E–H) With *moe* mutant, R8 clones did not terminate appropriately (arrow heads and enlarged insert in F). Note that axons of two R8 clones (indicated by arrows in F) joined below the lamina plexus and traveled together in the medulla. Their growth cones were extensively expanded and formed brush-like structures (enlarged insert in F). (I–L) With the *wgn* mutant, an axon of R8 clone projected into neighboring regions (arrow head and enlarged view in J). Panel L showed the entire eye-disc. Note that there were only two *wgn* mutant clones in entire eye-disc, one was R8 clone that was double labeled with anti-Senseless (indicated by the arrow heads in L and L′) and the other was not R8 clone (arrow in L and L′′). The R8 MARCM clones with anti-Senseless (blue) co-staining were indicated by arrowheads in D, H, and L. (M) Quantifications of the percentage of abnormal of R8 MARCM clones. Genotypes in M: *wt* (*FRT 19A*/*FRT 19A, Tub-Gal80, hsFLP*; *mCD8-GFP/+; rtl, GMR-Gal4*/+); *wgn* (*FRT 19A, wgn^22^*/*FRT 19A, Tub-Gal80, hsFLP*; *mCD8-GFP/+; rtl, GMR-Gal4*/+); *moe* (*FRT 19A, moe^G0323^*/*FRT 19A, Tub-Gal80, hsFLP*; *mCD8-GFP/+; rtl, GMR-Gal4*/+). Abbreviations: lp, lamina plexus; me, medulla. Scale bars: A–C, E–G, and I–K, 10 µm; D and H, 20 µm; L, 10 µm.

**Figure 8:**
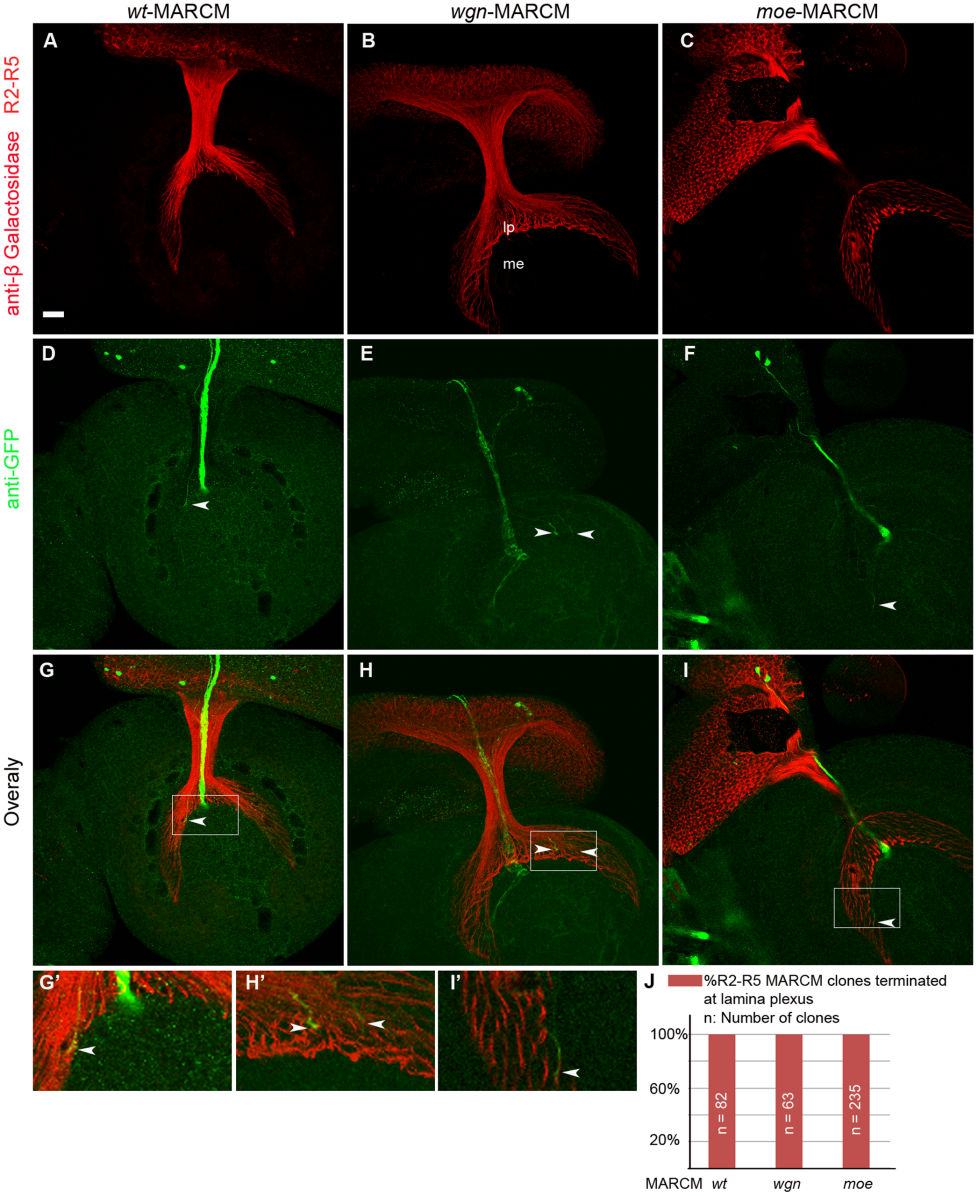
R2–R5 axonal targeting defects are non-cell autonomous. Axons of R2–R5 MARCM clones were induced by hs-FLP and labeled by GFP. (A–C) *Ro-tau-LacZ* labeled all the R2–R5 axons. (D–F) GFP labeled MARCM clones. (G–I) Overlay images of each genotype. (G′–I′) enlarged views of boxed areas in G–I. (D) Wild-type R2–R5 MARCM clone. (E) *wgn* mutant R2–R5 MARCM clones. (F) *moe* mutant R2–R5 MARCM clone. *wgn* or *moe* mutations in R2–R5 cells did not cause R2–R5 axon mistargeting and they terminated in the lamina plexus normally (arrow heads in D–I and G′–I′). (J) Quantifications of the targeting of R2–R5 MARCM clones at lamina plexus. Genotypes: *wt*(*FRT 19A*/*FRT 19A, Tub-Gal80, hsFLP*; *mCD8-GFP/+; rtl, GMR-Gal4*/+); *wgn*(*FRT 19A, wgn^22^*/*FRT 19A, Tub-Gal80, hsFLP*; *mCD8-GFP/+;rtl, GMR-Gal4*/+); *moe*(*FRT 19A, moe^G0323^*/*FRT 19A, Tub-Gal80, hsFLP*; *mCD-GFP/+; rtl, GMR-Gal4*/+). Abbreviations: lp, lamina plexus; me, medulla; *rtl*, *Rough-tau-LacZ*. Scale bar: 10 µm.

To determine if Wgn or Moe functions in a cell autonomous manner to control R cell axon targeting *in vivo*, we used the Mosaic Analysis with a Repressible Cell Marker (MARCM) method [20] to produce genetic mosaics that allowed us to follow axonal trajectories of individual wild-type or mutant R cell axons that were labelled with a mCD8-GFP fusion protein (Figure 7 and Figure 8). In wild-type animals, the R8 MARCM axons (GFP and Senseless positive) passed through the lamina to reach the medulla. In the medulla, they did not turn or occupy neighbouring target regions (arrow heads and enlarged picture in Figure 7B). In the lamina, individual wild-type R2–R5 MARCM axons (GFP and *Ro-tau-LacZ* positive) terminated in the lamina plexus as expected (double arrow heads in Figure 7C and arrow head in Figure 8D). Intriguingly, R8 MARCM axons with mutations in *wgn* or *moe* showed severe targeting defects (Figure 7F and 7J) whereas R2–R5 MARCM axons containing homozygous *wgn* or *moe* mutations showed appropriate targeting to the lamina region (Figure 8E–8F and 8H′–8I′). R8 MARCM axons lacking *moe* wandered off their normal trajectory and displayed unusually large growth cones with many fine processes (arrows and arrow heads in Figure 7F and enlarged insert). R8 MARCM axons lacking *wgn* did not stay in their appropriate target zone but instead strayed into neighboring regions (arrow heads in Figure 7J and enlarged insert). Figure 7M and Figure 8J show the quantifications of the percentages of abnormal projection and/or morphology of R8 MARCM clones and normal projection of R2–R5 MARCM clones, respectively. These MARCM analyses suggest that R8 targeting defects are cell autonomous whereas defects observed in R2–R5 targeting defects may be non-cell autonomous in *wgn* and *moe* mutants.

### R2–R5 Axon Targeting Defects in *moe* and *wgn* Mutants are Non-cell Autonomous

**Figure 9:**
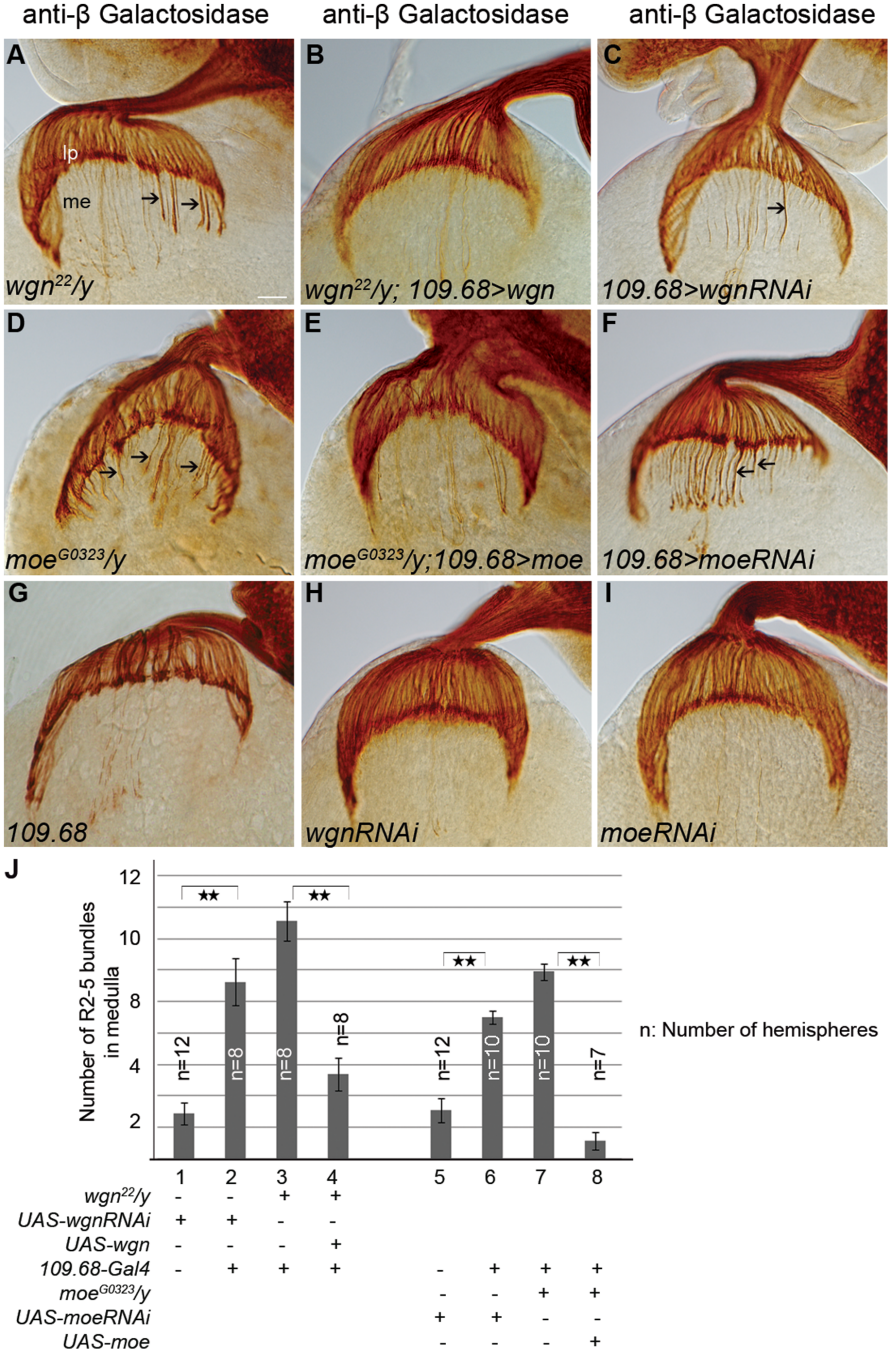
Wgn and Moe are required in R8 to regulate R2–R5 axonal targeting. Eye-brain complexes at third-instar larvae stage were labeled with *Ro-tau-LacZ* marker and stained by anti-beta galactosidase antibody. (A) *wgn* ^22^ */y* mutants showing R2–R5 mistargeting (arrows). (B) *wgn* ^22^/y mutants carrying one copy of *UAS-wgn* transgene under control of the R8 specific driver *109.68-Gal4* showed rescued R2–R5 axon mistargeting. (C) RNAi knockdown of Wgn only within R8 cells resulted in R2–R5 mistargeting into the medulla (axons >5) and/or thicker R2–R5 axonal bundles (arrow). (D) *moe^G0323^/y* mutants with R2–R5 mistargeting (arrows). (E) *moe^G0323^/y* mutants carrying one copy of *UAS-moe* transgene under control of the *109.68-Gal4* show substantial rescue. (F) RNAi knockdown of Moe within R8 resulted in R2–R5 axonal mistargeting (arrows). (G–I) Phenotypes were normal in larvae carrying *109.68-Gal4* driver (G), *UAS-wgn-RNAi* transgene (H) or *UAS-moe-RNAi* transgene (I). (J) Quantification of the number mistargeted of R2–R5 axon bundles presented in medulla in R8 specific knock down of Wgn or Moe or in R8 specific overexpression of Wgn or Moe in *wgn* or *moe* mutants, respectively. Double stars: P<0.001; Student T Tests, two tails. Error bars denote s.e.m. Genotypes in (J): #1(*wt^1118^*/*y*; *Bc*/+; *UAS-wgn-RNAi/rtl*); #2(*wt^1118^*/*y*; *109.68-Gal4*/+; *UAS-wgn-RNAi/rtl*); #3(*wgn^22^*/*y*; *109.68-Gal4*/+; *+/rtl*); #4(*wgn^22^*/*y*; *109.68-Gal4*/+; *UAS-wgn-Flag/rtl*; #5(*wt^1118^*/*y*; *Bc*/*UAS-moe-RNAi*; *rtl/+*); #6(*wt^1118^*/*y*; *109.68-Gal4*/*UAS-moe-RNAi; rtl/+*); #7(*moe^G0323^*/*y*; *109.68-Gal4*/*+; rtl*/+); 8#(*moe^G0323^*/*y*; *109.68-Gal4*/*UAS-moe-Myc; rtl*/+). Abbreviations: lp, lamina plexus; me, medulla; *rtl*, *Rough-tau-LacZ*. Scale bar: 10 µm.

R8 cells are the first differentiated R cells and R8 axons function as pioneers that help guide the axons of later developing R cells to their target zones [33]. Since our MARCM analyses indicated that *wgn* or *moe* mutations do not cause cell autonomous R2–R5 targeting defects (quantified in Figure 8J), we asked whether R2–R5 targeting defects observed in *wgn* and *moe* flies were secondary to the cell-autonomous defects in R8 axon targeting (Figure 9). To determine if Wgn expressed in R8 cells is required for normal R2–R5 targeting, we expressed Wgn or Moe in R8, but not other R cells, and assessed R2–R5 phenotypes in *wgn* or *moe* mutant animals, respectively. Strikingly, expression of a *UAS-wgn* transgene from an R8-specific driver (*Gal4^109–68^*) [17], [18] rescued R2–R5 mistargeting defects in a large proportion of *wgn^22^/y* individuals (66.7%; n = 18 animals; Figure 9B). R8-specific expression of the *UAS-wgn* transgene rescued the R2–R5 phenotypes considerably better than the neuronal expression of *UAS-wgn* driven by *Elav-Gal4* (66.7% versus 37.5%), supporting the hypothesis that R8-specific Wgn expression is required for normal R2–R5 targeting. Similarly, R8-specific expression of *UAS-moe* in an otherwise *moe*-null background rescued the R2–R5 phenotypes in 38% of individuals (n = 19 animals – see Figure 9E; Quantified in 9J). We conclude that activation of a Wgn-Moe signaling cassette within R8 cells restores normal R2–R5 targeting in mutant *wgn* or *moe* backgrounds and that this rescue relies upon the interaction of Moe with Wgn.

**Figure 10:**
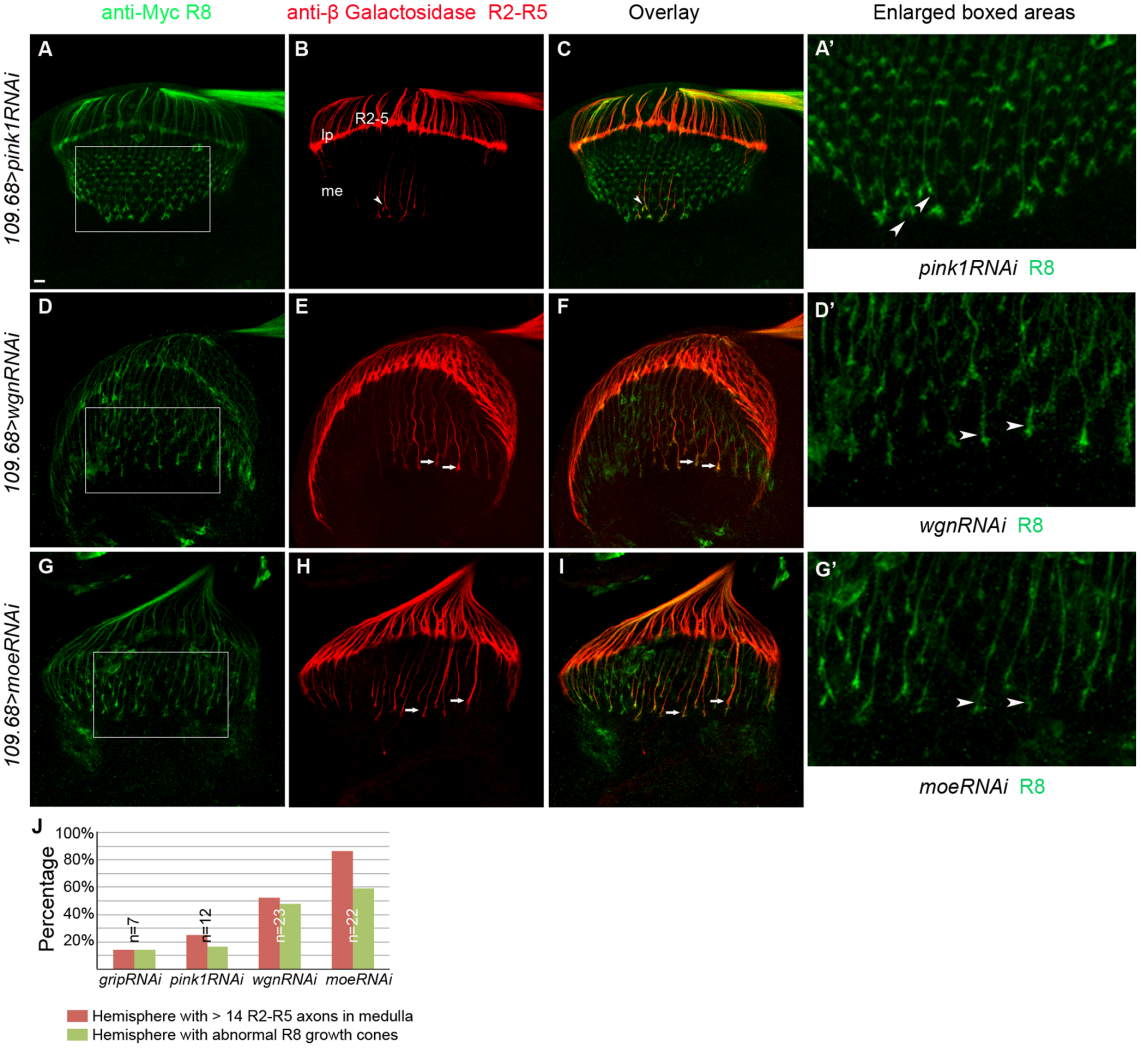
R8-psecific knockdown of Wgn or Moe results in cell-autonomous R8 growth cone defects and non-cell autonomous R2–R5 targeting defects. Samples were double labeled with *Ato-tau-Myc* for R8 (green) and *Ro-tau-LacZ* for R2–R5 (red). (A–C) R8 growth cone morphology and R2–R5 axon targeting were normal in control animals in which unrelated proteins (Pink1 or Grip) were specifically knocked down in R8 cells (*w^1118^*/*y*; *109.68-Gal4*/*UAS-pink1-RNAi; rtl, atm/+*. *w^1118^*/*y*; *109.68-Gal4*/ *UAS-grip-RNAi*; *rtl, atm/+*). The images showed R8 knock down of Pink1. (D–F) R8-specific knockdown of Wgn resulted in abnormal R8 growth cones (arrow heads in D′) in 47% of hemispheres and caused R2–R5 targeting defects in 56% of hemispheres (arrows in E; *w^1118^*/*y*; *109.68-GALl4*/+; *UAS-wgn-RNAi/rtl, atm*). (G–I) R8-specific knock down of Moe caused R8 growth cone morphology defects (arrow heads in G′) in 64% of hemispheres and R2–R5 mistargeting and/or bundled axons (arrows in H) in 86% of hemispheres (*w^1118^*/*y*; *109.68-Gal4*/*UAS-moe-RNAi; rtl, atm/+*). (A′, D′ and G′) Magnified views in boxed areas in A, D and G. (J) Quantifications. Hemispheres with > 14 R2–R5 axons and/or with thicker bundle in the medullar region were counted as hemispheres with R2–R5 mistargeting defects. Hemispheres that have irregular array of R8 growth cones in the medullar region with blunt or enlarged ends were counted as hemispheres with abnormal R8 growth cones. Abbreviations: lp, lamina plexus; me, medulla; *rtl*, *Rough-tau-LacZ*; *atm*, *Atonal-tau-Myc*. Scale bar: 10 µm.

**Figure 11:**
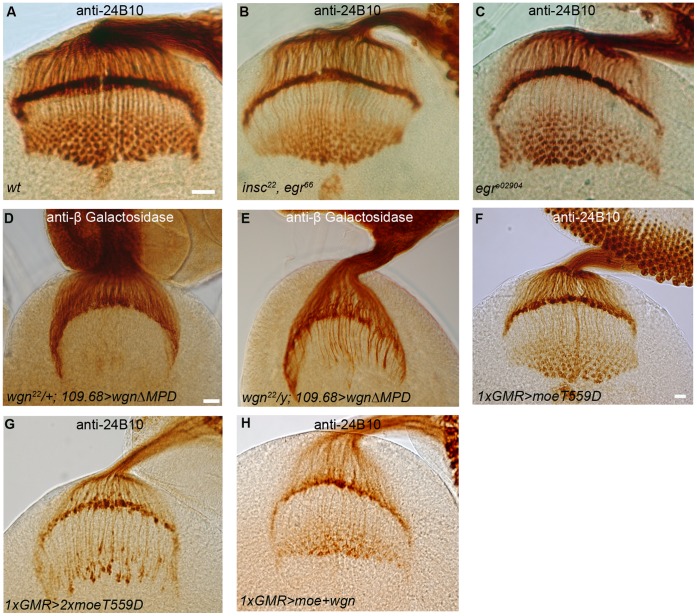
Analyses on R cell axon targeting in animals with different mutations. Samples in (A–C) and (F–H) were stained with 24B10. Samples in (D) and (E) were stained with anti-beta galactosidase. (A) Wild-type. (B) *insc^22^*, *egr^66^* (n = 8 animals). (C) *egr^e02904^* (n = 9 animals). Axonal targeting and growth cone morphology were normal in *egr* mutants. (D) Control. The termination of R2–R5 axons was not affected when a *UAS-wgn-ΔMPD* transgene was overexpressed in R8 cells (*wgn^22^/+; 109.68-Gal4/UAS-wgn-ΔMPD-Flag; rtl/+*). (E) 76% *wgn* mutants still presented mistargeted R2–R5 axons in the medulla region when a *UAS-wgn-ΔMPD* transgene was expressed in R8 cells (*wgn^22^/y; 109.68-Gal4/UAS-wgn-ΔMPD-Flag; rtl/+).* (F) Overexpression of one copy of *UAS-moeT559D* with *GMR-Gal4.* The lamina plexus was uneven. (G) Overexpression of two copies of *UAS-moeT559* with one copy *GMR-Gal4*. The targeting of R cell axons was severely disrupted with many clumps presented in lamina plexus and irregular thick bundles in medullar. (H) Overexpression of one copy of *UAS-moe* and one copy of *UAS-wgn* with *GMR-Gal4*. Abbreviation: *rtl*, *Rough-tau-LacZ.* Scale bars: A–C, 10 µm; D–E, 10 µm; F–H, 10 µm.

To further determine whether Wgn and Moe expression in R8 cells is required for R2–R5 axon targeting, RNA interference was used to reduce levels of Wgn or Moe only within R8 cells, of otherwise wild-type animals. In flies in which R8 cells were marked by *Ato-tau-Myc* and R2–R5 were marked with *Ro-tau-LacZ,* R8-specific expression of control *RNAi* transgenes (*UAS-pink1-RNAi* or *UAS-grip-RNAi*) had no effect on targeting of R8 or of R2–R5 axons (Figure 10A–10C and 10A′). However, R8-specific expression of *UAS-wgn-RNAi* not only resulted in the expected cell-autonomous defect in R8 axon targeting and growth cone morphology but also caused R2–R5 axonal targeting defects that were very similar to those observed in whole *wgn* mutant animals (Figure 9, Figure 10D–10F and 10D′). Similarly, R8-specific knockdown of Moe caused cell autonomous defects in R8 cells and non-cell autonomous defects in R2–R5 cells (Figure 9, Figure 10G–10I and 10G′). Quantifications of the number of R2–R5 axons entering the medulla (Figure 9J) and the penetrance of the R8 and R2–R5 phenotypes (Figure 10J) show that R8 specific expression Wgn or Moe partially rescues the R2–R5 mistargeting defects and loss of Wgn or Moe expression specifically in R8 caused not only the abnormal R8 growth cone phenotypes but also the profound R2–R5 axon mistargeting defects. Furthermore, R8 expression of the Wgn mutant protein that lacked the Moe binding motif produced only modest rescue (24%; n = 37 hemispheres; Figure 11), suggesting the interaction between Wgn and Moe in R8 is important for Wgn mediating signaling to control R cell axon targeting. We conclude that the Wgn-Moe signaling cassette regulates both cell autonomous R8 growth cone targeting and non-cell autonomous R2–R5 axon targeting.

## Discussion

Determining how guidance receptors transmit signals to regulate precise path finding decisions is crucial for understanding the development of nervous system. Here, we have identified a Wgn-Moe signaling cascade that plays important roles in regulating R cell axon targeting during development. Several lines of evidence support this contention. First, mutations in *wgn* or *moe* cause severe R cell axon targeting defects. Second, like Moe, Wgn is expressed in R cells, predominantly in R8. Third, Wgn physically interacts with Moe through the MPD region present in the receptor’s intracellular domain; this interaction is necessary for its ability to regulate R cell axon targeting. Fourth, reducing the gene dosage of *moe* by half enhances the R cell axon mistargeting phenotypes in *wgn* hypomorphic animals. Importantly, we also report that mutations in *wgn* or *moe* cause R8 growth cone morphology and mistargeting defects that are cell-autonomous and R2–R5 axon mistargeting defects that are non cell-autonomous. We therefore propose that the Wgn-Moe signaling cascade contributes to R cell axon targeting via two distinct mechanisms: first, Wgn-Moe acts as a receptor complex in R8 to regulate morphology of R8 growth cones and to guide proper targeting and second, this cascade acts to prevent R2–R5 axons from mistargeting into the medulla.

The proposed cell-autonomous role of Wgn-Moe signaling cascade in R8 cells is supported by three key findings. First, Wgn is expressed predominately in R8. Second, selective mutation of *wgn* or *moe* only in R8 by MARCM, results in abnormally expanded and mistargeted R8 growth cones. Third, R8-specific knockdown of Wgn or Moe by using *RNAi* lines causes R8 growth cone targeting and morphology defects.

When growth cones reach their appropriate target, they condense and their actin cytoskeleton changes from a very dynamic structure to a stabilized actin network that supports newly formed cell-cell contacts. One of the functions of ERM proteins is to bring receptors in close proximity to downstream signaling components required to alter the actin cytoskeleton. We speculate that in R8 cells, Moe acts as an adaptor that allows environmental signals sensed by Wgn to alter actin cytoskeleton dynamics and thus transduce a stop signal that halts R8 axon extension. Candidates that lie downstream of Moe in this pathway are unknown but small GTPases seem likely to play a role. TNF/TNFR superfamily members function as potent regulators of small GTPases in mammals [9], [34] and in flies, Moe can negatively regulate Rho1 activity [28]. It is notable that overexpression of a dominant-negative form of Rho1 in R cells causes axon guidance defects that resemble those in flies overexpressing a constitutively active Moe (*UAS-moeT559D*) [28], [35](Figure 11).

How is the Wgn-Moe signal cascade activated? Egr is the only TNF-like ligand in *Drosophila* and previous studies have shown that it functions as a ligand for Wgn. When overexpressed in the developing eye, Egr induces cell death through a Wgn-dependent pathway and more recent work suggests physiological roles for the Egr-Wgn signaling cascade in tumour suppression and in regulation of pain responses [10], [12], [13], [36]–[38]. Given the well established lines between Egr and Wgn, we were surprised to find that neither of the two distinct *egr* mutant lines examined in this study had defects in R cell axon targeting (Figure 11B and 11C). Thus, Wgn regulates R cell axon targeting in an Egr-independent manner, indicating that Wgn must bind alternate ligands or function in a ligand-independent manner. We favour the latter hypothesis since R cell axon targeting defects caused by Wgn overexpression in the eye were not enhanced when the dosage of the *UAS-wgn* transgene was increased whereas increasing *UAS-moe* overexpression had a drastic effect (quantified in Figure 6N and 6O). In addition, overexpression of constitutive active form of the Moe (*UAS-moeT559D*) in fly eye leads to severe R cell axon targeting defects at third-instar larval stage (Figure and 11F and 11G) and causes a rough eye phenotype at the adult stage (data not shown) in a dosage dependent manner whereas overexpression of *UAS-wgn* and *UAS-moe* together did not (Figure 11H). These findings suggest that a change in Wgn signaling properties, most likely mediated by an activating ligand, may regulate axonal targeting.

Three findings support the conclusion that Wgn-Moe signaling in R8 photoreceptors functions to regulate R2–R5 axon targeting in a non cell-autonomous manner. First, global deletion of Wgn or Moe causes R2–R5 axons mistargeted into medulla whereas individually mutated R2–R5 axons of *wgn* or *moe* target correctly. Second, R8-specific knockdown of Wgn or Moe leads to R2–R5 axon mistargeting defects. Third, expression of Wgn or Moe specifically in R8 cells rescues the R2–R5 mistargeting defects. The R8-dependent regulation of R2–R5 targeting reported here is, to our knowledge, a novel finding that is unique for the Wgn-Moe signaling cassette. Golden Goal and Flamingo proteins have been shown to alter R8 and R1-6 targeting but available data indicate that these are due solely to cell autonomous events [25], [26], [39], [40].

How does Wgn-Moe signaling within R8 cells facilitate correct targeting of R2–R5 axons? One possibility is that the Wgn-Moe signaling cascade may regulate expression or function of R8 cell surface molecule(s) that function(s) as a stop signal for R1-6 axons. There is no direct evidence for Wgn itself acting as a stop signal but other TNFR superfamily members can impact on growth inhibitory molecules. In mammals, p75^NTR^ and Troy, both mammalian TNFR superfamily members, transduce stop-growth signals mediated by the Nogo receptor [41], [42]. p75^NTR^ has also been shown to functionally interact with the Semaphorin3 receptors, Neuropilin1 and PlexinA4 [43]. In fly, PlexinA regulates R cell axon guidance [35] and it is conceivable that Wgn and /or Moe may interact with this pathway. Alternatively, the Wgn-Moe signaling cascade may produce a soluble factor that drives expression of a stop signal within R2–R5 cells or in cells that they contact in their target zone. Identifying the nature of the non cell-autonomous signal(s) produced by R8 cells to regulate R2–R5 targeting will be an interesting challenge for future studies.

To summarize, we have shown that the sole TNFR and ERM protein in fly functionally and physically interact with each other to regulate R cell axonal targeting during development. The Wgn-Moe signaling cassette functions in a cell autonomous manner to ensure that R8 axons respond to layer specific termination signals and functions non-cell autonomously to regulate R2–R5 layer recognition.

## References

[1] Tessier-Lavigne M, Goodman CS (1996) The molecular biology of axon guidance. Science 274: 1123–1133. 8895455 10.1126/science.274.5290.1123

[2] Sanes JR, Zipursky SL (2010) Design principles of insect and vertebrate visual systems. Neuron 66: 15–36. 20399726 10.1016/j.neuron.2010.01.018 PMC2871012

[3] Meinertzhagen I, Hanson TE (1993) The development of the optic lobe. In The Development of Drosophila melanogaster, A. Martinez-Arias and M. Bate, eds. New York: Cold Spring Harbor Press. 1363–1490.

[4] Wolff T, Martin KA, Rubin GM, Zipursky SL (1997) The development of the Drosophila visual system. In Molecular and Cellular Approaches to Neural Development and S.L. Zips intra- 474–508. eds. New York Oxford University Press 474–508.

[5] Mathew SJ, Haubert D, Kronke M, Leptin M (2009) Looking beyond death: a morphogenetic role for the TNF signalling pathway. J Cell Sci 122: 1939–1946. 19494121 10.1242/jcs.044487

[6] Zuliani C, Kleber S, Klussmann S, Wenger T, Kenzelmann M, et al (2006) Control of neuronal branching by the death receptor CD95 (Fas/Apo-1). Cell Death Differ 13: 31–40. 16003386 10.1038/sj.cdd.4401720

[7] Nikolaev A, McLaughlin T, O’Leary DD, Tessier-Lavigne M (2009) APP binds DR6 to trigger axon pruning and neuron death via distinct caspases. Nature 457: 981–989. 19225519 10.1038/nature07767 PMC2677572

[8] Desbarats J, Birge RB, Mimouni-Rongy M, Weinstein DE, Palerme JS, et al (2003) Fas engagement induces neurite growth through ERK activation and p35 upregulation. Nat Cell Biol 5: 118–125. 12545171 10.1038/ncb916

[9] Ruan W, Lee CT, Desbarats J (2008) A novel juxtamembrane domain in tumor necrosis factor receptor superfamily molecules activates Rac1 and controls neurite growth. Mol Biol Cell 19: 3192–3202. 18508927 10.1091/mbc.E08-02-0161 PMC2488301

[10] Kanda H, Igaki T, Kanuka H, Yagi T, Miura M (2002) Wengen, a member of the Drosophila tumor necrosis factor receptor superfamily, is required for Eiger signaling. J Biol Chem 277: 28372–28375. 12084706 10.1074/jbc.C200324200

[11] Kauppila S, Maaty WS, Chen P, Tomar RS, Eby MT, et al (2003) Eiger and its receptor, Wengen, comprise a TNF-like system in Drosophila. Oncogene 22: 4860–4867. 12894227 10.1038/sj.onc.1206715

[12] Igaki T, Kanda H, Yamamoto-Goto Y, Kanuka H, Kuranaga E, et al (2002) Eiger, a TNF superfamily ligand that triggers the Drosophila JNK pathway. EMBO J 21: 3009–3018. 12065414 10.1093/emboj/cdf306 PMC126061

[13] Moreno E, Yan M, Basler K (2002) Evolution of TNF signaling mechanisms: JNK-dependent apoptosis triggered by Eiger, the Drosophila homolog of the TNF superfamily. Curr Biol 12: 1263–1268. 12176339 10.1016/s0960-9822(02)00954-5

[14] Fehon RG, McClatchey AI, Bretscher A (2010) Organizing the cell cortex: the role of ERM proteins. Nat Rev Mol Cell Biol 11: 276–287. 20308985 10.1038/nrm2866 PMC2871950

[15] McCartney BM, Fehon RG (1996) Distinct cellular and subcellular patterns of expression imply distinct functions for the Drosophila homologues of moesin and the neurofibromatosis 2 tumor suppressor, merlin. J Cell Biol 133: 843–852. 8666669 10.1083/jcb.133.4.843 PMC2120840

[16] Wang H, Cai Y, Chia W, Yang X (2006) Drosophila homologs of mammalian TNF/TNFR-related molecules regulate segregation of Miranda/Prospero in neuroblasts. EMBO J 25: 5783–5793. 17139248 10.1038/sj.emboj.7601461 PMC1698905

[17] Jarman AP, Ahmed I (1998) The specificity of proneural genes in determining Drosophila sense organ identity. Mech Dev 76: 117–125. 9767145 10.1016/s0925-4773(98)00116-6

[18] Chien CT, Hsiao CD, Jan LY, Jan YN (1996) Neuronal type information encoded in the basic-helix-loop-helix domain of proneural genes. Proc Natl Acad Sci U S A 93: 13239–13244. 8917575 10.1073/pnas.93.23.13239 PMC24077

[19] Thibault ST, Singer MA, Miyazaki WY, Milash B, Dompe NA, et al (2004) A complementary transposon tool kit for Drosophila melanogaster using P and piggyBac. Nat Genet 36: 283–287. 14981521 10.1038/ng1314

[20] Lee T, Luo L (1999) Mosaic analysis with a repressible cell marker for studies of gene function in neuronal morphogenesis. Neuron 22: 451–461. 10197526 10.1016/s0896-6273(00)80701-1

[21] Ruan W, Pang P, Rao Y (1999) The SH2/SH3 adaptor protein dock interacts with the Ste20-like kinase misshapen in controlling growth cone motility. Neuron 24: 595–605. 10595512 10.1016/s0896-6273(00)81115-0

[22] Van Vactor DL Jr, Cagan RL, Kramer H, Zipursky SL (1991) Induction in the developing compound eye of Drosophila: multiple mechanisms restrict R7 induction to a single retinal precursor cell. Cell 67: 1145–1155. 1760842 10.1016/0092-8674(91)90291-6

[23] Fujita SC, Zipursky SL, Benzer S, Ferrus A, Shotwell SL (1982) Monoclonal antibodies against the Drosophila nervous system. Proc Natl Acad Sci U S A 79: 7929–7933. 6818557 10.1073/pnas.79.24.7929 PMC347463

[24] Garrity PA, Lee CH, Salecker I, Robertson HC, Desai CJ, et al (1999) Retinal axon target selection in Drosophila is regulated by a receptor protein tyrosine phosphatase. Neuron 22: 707–717. 10230791 10.1016/s0896-6273(00)80730-8

[25] Tomasi T, Hakeda-Suzuki S, Ohler S, Schleiffer A, Suzuki T (2008) The transmembrane protein Golden goal regulates R8 photoreceptor axon-axon and axon-target interactions. Neuron 57: 691–704. 18341990 10.1016/j.neuron.2008.01.012

[26] Senti KA, Usui T, Boucke K, Greber U, Uemura T, et al (2003) Flamingo regulates R8 axon-axon and axon-target interactions in the Drosophila visual system. Curr Biol 13: 828–832. 12747830 10.1016/s0960-9822(03)00291-4

[27] Nolo R, Abbott LA, Bellen HJ (2000) Senseless, a Zn finger transcription factor, is necessary and sufficient for sensory organ development in Drosophila. Cell 102: 349–362. 10975525 10.1016/s0092-8674(00)00040-4

[28] Speck O, Hughes SC, Noren NK, Kulikauskas RM, Fehon RG (2003) Moesin functions antagonistically to the Rho pathway to maintain epithelial integrity. Nature 421: 83–87. 12511959 10.1038/nature01295

[29] Karagiosis SA, Ready DF (2004) Moesin contributes an essential structural role in Drosophila photoreceptor morphogenesis. Development 131: 725–732. 14724125 10.1242/dev.00976

[30] Freeman M (1996) Reiterative use of the EGF receptor triggers differentiation of all cell types in the Drosophila eye. Cell 87: 651–660. 8929534 10.1016/s0092-8674(00)81385-9

[31] Robinow SWK (1991) Characterizationand spatial distribution of the ELAV protein during Drosophila melanogaster development. J Neurobiol 22: 443–461. 1716300 10.1002/neu.480220503

[32] Martin-Blanco E, Gampel A, Ring J, Virdee K, Kirov N, et al (1998) puckered encodes a phosphatase that mediates a feedback loop regulating JNK activity during dorsal closure in Drosophila. Genes & Development 12: 557–570. 9472024 10.1101/gad.12.4.557 PMC316530

[33] White NM, Jarman AP (2000) Drosophila atonal controls photoreceptor R8-specific properties and modulates both receptor tyrosine kinase and Hedgehog signalling. Development 127: 1681–1689. 10725244 10.1242/dev.127.8.1681

[34] Neumann H, Schweigreiter R, Yamashita T, Rosenkranz K, Wekerle H, et al (2002) Tumor necrosis factor inhibits neurite outgrowth and branching of hippocampal neurons by a rho-dependent mechanism. J Neurosci 22: 854–862. 11826115 10.1523/JNEUROSCI.22-03-00854.2002 PMC6758473

[35] Yu L, Zhou Y, Cheng S, Rao Y (2010) Plexin a-semaphorin-1a reverse signaling regulates photoreceptor axon guidance in Drosophila. J Neurosci 30: 12151–12156. 20826677 10.1523/JNEUROSCI.1494-10.2010 PMC6633550

[36] Babcock DT, Shi S, Jo J, Shaw M, Gutstein HB, et al (2011) Hedgehog signaling regulates nociceptive sensitization. Curr Biol 21: 1525–1533. 21906949 10.1016/j.cub.2011.08.020 PMC3262399

[37] Babcock DT, Galko MJ (2009) Two sides of the same coin no longer: genetic separation of nociceptive sensitization responses. Commun Integr Biol 2: 517–519. 20195458 10.4161/cib.2.6.9561 PMC2829827

[38] Igaki T, Pastor-Pareja JC, Aonuma H, Miura M, Xu T (2009) Intrinsic tumor suppression and epithelial maintenance by endocytic activation of Eiger/TNF signaling in Drosophila. Dev Cell 16: 458–465. 19289090 10.1016/j.devcel.2009.01.002 PMC2729686

[39] Hakeda-Suzuki S, Berger-Muller S, Tomasi T, Usui T, Horiuchi SY, et al (2011) Golden Goal collaborates with Flamingo in conferring synaptic-layer specificity in the visual system. Nat Neurosci 14: 314–323. 21317905 10.1038/nn.2756

[40] Lee RC, Clandinin TR, Lee CH, Chen PL, Meinertzhagen IA, et al (2003) The protocadherin Flamingo is required for axon target selection in the Drosophila visual system. Nat Neurosci 6: 557–563. 12754514 10.1038/nn1063

[41] Wang KC, Kim JA, Sivasankaran R, Segal R, He Z (2002) P75 interacts with the Nogo receptor as a co-receptor for Nogo, MAG and OMgp. Nature 420: 74–78. 12422217 10.1038/nature01176

[42] Park JB, Yiu G, Kaneko S, Wang J, Chang J, et al (2005) A TNF receptor family member, TROY, is a coreceptor with Nogo receptor in mediating the inhibitory activity of myelin inhibitors. Neuron 45: 345–351. 15694321 10.1016/j.neuron.2004.12.040

[43] Ben-Zvi A, Ben-Gigi L, Klein H, Behar O (2007) Modulation of semaphorin3A activity by p75 neurotrophin receptor influences peripheral axon patterning. J Neurosci 27: 13000–13011. 18032673 10.1523/JNEUROSCI.3373-07.2007 PMC6673287

